# Does the predominance of central sensitization alter the effect of balneotherapy combined with conventional physical therapy in patients with chronic low back and knee pain?

**DOI:** 10.1007/s00484-026-03157-w

**Published:** 2026-02-24

**Authors:** Dilek Ulusoy Özkan, Musa Polat

**Affiliations:** Department of Physical Medicine and Rehabilitation, Faculty of Medicine, Sivas Cumhuriyet University, Sivas, Turkey

**Keywords:** Balneotherapy, central sensitization, chronic pain, physical therapy

## Abstract

Various methods, including balneotherapy, are employed in the management of patients with chronic pain. This research investigated the effect of the predominance of central sensitization on responses to balneotherapy applied together with conventional physical therapy in patients with chronic low back and knee pain. This prospective study included 75 patients with chronic low back or knee pain, classified as having predominant central sensitization (*n* = 35) or not (*n* = 40). All participants received a three-week course of whole-body balneotherapy combined with conventional physical therapy, including transcutaneous electrical nerve stimulation, hot pack, and therapeutic ultrasound. Pain intensity, as the primary outcome, was assessed using the visual analogue scale (VAS), while quality of life and emotional status were evaluated using the Short Form-12 (SF-12) and the Hospital Anxiety and Depression Scale (HADS), respectively. Disability associated with chronic pain was evaluated using the Roland Morris Disability Questionnaire in patients with low back pain and the Knee Injury and Osteoarthritis Outcome Score (KOOS) in those with knee pain. Changes in outcomes over time and between groups were analyzed using linear mixed-effects models. Improvement in the VAS, SF-12 Physical and Mental Component, HADS-Depression, and HADS-Anxiety scores was observed in both groups post-treatment (between *p* < 0.01 and *p* = 0.015). Also, post-treatment improvement in disability scores was observed in both groups (between *p* < 0.01 and 0.021). Group × time interaction was observed only for the SF-12 mental component score, in favor of the predominant central sensitization group (ΔΔ = 5.95, 95% CI 1.31 to 10.59, *p* = 0.013). No significant group × time interaction was observed for other outcomes (range: 0.10–0.97). Applied together with physical therapy agents with balneotherapy can reduces pain severity and disability levels, and increases their quality of life in patients with chronic pain with and without predominant central sensitization. Improvements in mental health–related quality of life were more pronounced in patients with predominant central sensitization.

## Introduction

Chronic pain is a complex condition characterized by the persistence of pain after the effect of a nociceptive stimulus has ceased, altering the individual’s quality of life, and having no discernible biological purpose (Mills et al. 2019). Changes in neuroendocrine function and sympathetic tone may be seen in patients with chronic pain, physical or mental complaints develop, and unpleasant complex experiences commence. Disability due to chronic pain eventually occurs, restricting individuals’ ability to perform essential activities (Kamper et al. 2015). Symptoms and signs observed in patients with chronic pain, such as exhaustion, fatigue, sleep disorder, depression, lack of appetite, weight loss, increased irritability, decreased libido and sexual activity, constipation, psychomotor disorders, and communication problems in patients with chronic pain define chronic pain as a syndrome (Breivik et al. 2006; Cohen et al. 2021; Nugraha et al. 2019). Neuronal plasticity in the pain coding pathways, mechanisms resulting from peripheral sensitization in the dorsal root ganglia and primary sensory neurons and, more importantly, central sensitization in pain processing neurons in the spinal cord and brain are thought to be responsible for this entire process (Ossipov et al. 2010; Woolf 2011).

Central sensitization occurs when nociceptive receptors in the central nervous system increase their capacity to respond to sub-threshold stimuli (Song et al. 2024). N-methyl-D-aspartate (NMDA) receptor activation (Ji et al. 2018), neuroplasticity, impaired function in descending inhibitory pathways (Ossipov et al. 2014), and neuroinflammation all play a role in central sensitization. In the presence of central sensitization, there is overactivity of pain-facilitating pathways and dysfunction of pain-inhibiting pathways (Meeus and Nijs 2007). This is adaptive at the onset of pain and contributes to the re-acquisition of the body’s homeostatic control. However, if the process continues it becomes maladaptive and results in the persistence of symptoms without tissue damage and in the development of several psychosomatic symptoms (Nijs et al. 2019). Even in chronic pain in which nociceptive input from peripheral tissues continues, central sensitization is reported to exert an effect independent of peripheral events (Imamura et al. 2008; Staud 2011). For that reason, central sensitization must be considered in the management of chronic pain, and factors such as anxiety, depression, pain catastrophization, physical immobility, and sleep disorders must also be borne in mind (Nijs et al. 2023). Indeed, patients with central sensitization have been reported to experience more severe pain, greater disability, higher levels of depression and anxiety, and poorer quality of life. (Smart et al. 2012).

These recent developments in the field of neuroscience have altered perspectives toward chronic pain, and have revealed the need to investigate the presence of central sensitization in the management of chronic pain. This awareness has raised the question of whether physical therapy modalities, non-pharmacological agents employed in the management of chronic pain, exert different effects on patients with chronic pain with and without central sensitization. In this context, in their randomized controlled research involving participants with low back pain, Leemans et al. (2021) showed that a combination of transcutaneous electrical nerve stimulation (TENS) and superficially applied heat did not affect on pain inhibition function, and these agents have been interpreted as acting via local effects rather than through brain-mediated analgesic ones (Leemans et al. 2021). Yüzügüldü et al. (2023) reported higher central sensitization scores in a group that did not respond to traditional physical therapy consisting of TENS, ultrasound, and hot pack among patients with chronic pain associated with knee osteoarthritis (Yüzügüldü et al. 2023). Few studies have evaluated the effect of physical therapy agents in the presence of central sensitization, and research involving different non-pharmacological methods is needed.

Balneotherapy, used as a well-tolerable physical therapy agent in chronic pain management, is known to improve pain scores and psychosocial well-being (Roques and Queneau 2016). Its efficacy can be explained through different mechanisms. In general terms, the temperature and buoyancy of the water reduce pain and muscular stiffness by exerting a positive effect on muscle tone (Silva et al. 2023). However, studies have reported that balneotherapy balances the levels of antioxidants and free oxygen radicals thought to play a role in the pathogenesis of central sensitization and hyperalgesia (Çetinkaya et al. 2020), and that the thermal spa setting is important with its effects on functional mobility and a healthy lifestyle, in addition to physical therapy (Silva et al. 2023).

To our best knowledge, no previous studies have investigated the role of central sensitization in the effect of balneotherapy, capable of being used alone or together with other modalities in the management of chronic pain. The aim of this study was to compare the effects of a combination of balneotherapy and physical therapy on pain, disability, quality of life, and mood in patients with chronic knee and low back pain with and without predominant central sensitization.

## Materıal and methods

### Participants

In this prospective comparative study, 75 patients aged ≥ 18 years with knee or low back pain lasting longer than three months and a visual analogue scale (VAS) pain score of ≥ 4 were consecutively included. All patients were treated as inpatients at the Sivas Cumhuriyet University Health Services Application and Research Sıcak Çermik Physical Therapy Center, Türkiye, between 1 January and 31 July 2024. Chronic low back pain and chronic knee pain are among the most prevalent musculoskeletal pain conditions encountered in clinical practice. Although these conditions differ anatomically, both frequently exhibit features of central sensitization, particularly in chronic stages (Nijs et al. 2019; Staud 2011). This shared pathophysiological background, these conditions provide a suitable clinical model for examining the influence of central sensitization on treatment response. Therefore, patients with chronic low back pain and chronic knee pain were jointly included in the present study.

Patients were excluded if they had a history of lumbar or knee surgery; central or peripheral nervous system disorders; uncontrolled hypertension; inflammatory rheumatological diseases; fibromyalgia; organ failure; malignancy; infectious or metabolic diseases; or psychiatric disorders. In addition, patients who had received physical therapy modalities, invasive pain-related procedures within the previous six months, or pharmacological treatment for chronic pain were excluded.

The participants were informed about the scope and aim of the research and provided written and verbal consent to take part. The study protocol and informed consent form had previously been approved by the local ethical committee (no. 2023-12/55), and the research was conducted in compliance with the Declaration of Helsinki and good clinical practice guidelines.

### Evaluation

The participants’ sociodemographic and clinical data, including age, sex, education level, occupation, height, weight, body mass index (BMI), systemic diseases, drugs used, and duration of symptoms, were recorded using a standardized form. Pain levels were evaluated using a 100-mm VAS, quality of life using the Short Form-12 Quality of Life Scale (SF-12), mood using the Hospital Anxiety and Depression Scale (HADS), and disability levels using the Roland Morris Disability Questionnaire (RMDQ) in patients with low back pain and the Knee Injury and Osteoarthritis Outcome Score (KOOS) in patients with knee pain. The primary outcome of the study was VAS. Secondary outcomes included SF-12, HADS, RMDQ and KOOS.

The Short Form-12 Quality of Life Scale (SF-12) is a comprehensive tool that encapsulates eight distinct domains through 12 insightful items. Respondents answer the items concerning physical and emotional role limitations with a simple “yes” or “no,” while the remaining questions utilize a nuanced Likert-type scale ranging from 3 to 6. From these responses, a Physical Component Summary (PCS-12) score emerges, drawing from general health, physical functioning, role limitations due to physical health challenges, and bodily pain. Meanwhile, the Mental Component Summary (MCS-12) score encompasses dimensions of social functioning, role limitations attributed to emotional issues, mental health, and energy levels. Both PCS-12 and MCS-12 scores span from 0 to 100, where higher scores reflect superior health and well-being. The Turkish version of the SF-12 stands as a robust measurement tool, showcasing exceptional psychometric properties for evaluating general health status and health-related quality of life in both clinical and research environments (Soylu and Kütük 2022).

The Hospital Anxiety and Depression Scale consists of 14 items, seven investigating anxiety subscale symptoms and seven depression subscale symptoms on a four-point Likert-type scale ranging between 0 and 3. Rather than being diagnostic, the purpose of the scale is to identify risk groups and evaluate changes in the patient’s emotional stats. The reliability and validity of the Turkish-language version of HADS have previously been established (Aydemir and Küey 1997).

The Roland-Morris Disability Questionnaire was developed to evaluate functional impairment in patients with low back pain. It consists of 24 questions related to functional impairment, with patients being asked to respond ‘yes’ if a statement applies to them, or ‘no’ if not. Positive responses are scored 1, and negative responses 0. Total possible scores range between 0 and 24, with higher scores indicating greater disability (Dilekçi et al. 2020).

The Knee Injury and Osteoarthritis Outcome Score serves to evaluate symptoms and functional status associated with knee injuries and osteoarthritis. It consists of 42 items in five subscales, symptoms and stiffness, pain, activities of daily living, functional status in sport and recreation, and knee-related quality of life. It assesses both the short- and long-term outcomes of knee injuries. The Turkish-language version has previously been validated (Paker et al. 2007).

### Groups

Before the first balneotherapy session, the predominance of central sensitization features was evaluated by a researcher blinded to the outcome measure findings, in accordance with a previously defined, multidimensional assessment protocol (Nijs et al. 2014). Within this framework, participants were assessed based on clinical history, physical examination findings, and available laboratory and radiological investigations. Participants with pain disproportionate to identifiable structural pathology, widespread pain distribution, clinical signs of allodynia and/or hyperalgesia, and Central Sensitization Inventory (CSI) scores above 40 were grouped as patients with predominant central sensitization (Neblett et al. 2013).

The CSI was developed to provide a better evaluation of symptoms thought to be related to central sensitization, to categorize symptoms, to define the disease severity, and to plan patient management. Part A is used to identify central sensitization by investigating 25 symptoms. Possible scores on this four-point Likert-type scale range between 0 and 100. Scores exceeding 40 are regarded as indicating the presence of central sensitization (Neblett et al. 2013). A higher total indicates greater symptom severity. Part B of the CSI evaluates whether a patient has previously been diagnosed with one or more conditions associated with central sensitization and is not scored. The Turkish-language version of the CSI is employed to screen with high specificity and sensitivity for the presence of central sensitization in patients presenting with chronic pain (Düzce Keleş et al. 2021).

### Intervention

Balneotherapy sessions were performed using the full body method with natural spring water possessing thermomineral water characteristics according to the International Society of Medical Hydrology and Climatology (water temperature 38–40° C, contents: calcium 445.5 mg/L, sodium 215.8 mg/L, chloride 214.8 mg/L, magnesium 74.5 mg/L, sulfate 53.1 mg/L, sulfur 9.2 mg/L, iron 3.14 mg/L, and iodide 0.52 mg/L) at the Cumhuriyet University physical therapy center in Sıcak Çermik in Sivas, one of the leading thermal centers in Türkiye. Participants underwent daily balneotherapy sessions for 21 consecutive days, each lasting 10 min, under the supervision of a physiatrist and a nurse. Balneotherapy was administered in a thermal pool with a depth of 1.5 m and was conducted exclusively as passive immersion; no aquatic exercises were incorporated during the sessions. In addition, traditional physical therapy applications such as hot packs, TENS, and therapeutic continuous ultrasound were applied by a trained technician to the primary pain area (lower back or knee) on the same day either before or after the balneotherapy session as part of a standardized protocol.

Therapeutic continuous ultrasound was applied to the lumbar paravertebral region of the patients with chronic low back pain and to the knee joints of those with chronic knee pain for six minutes at a frequency of 1 MHz and an intensity of 1.6 W/cm 2 using a BTL-4000 Premium device (BTL Medical Technologies Pty Ltd, Australia). TENS was applied for 20 min using a Fizyomed Fizyotens 4000 device (Fizyomed Tıbbi Cihazlar Ltd., Türkiye) in conventional flow mode, in a 60–100 current frequency range, at the amplitude at which the patient experienced a tingling sensation, using electrodes attached vertically to the lumbar paravertebral region of patients with chronic low back pain and to the lateral and medial patella of those with chronic knee pain. The hot packs, kept in 60° C heaters, were removed and wrapped in the patients’ towels in order to prevent skin contact and were applied to the lumbar or knee regions for 20 min.

### Statistical analysis

The sample size was determined based on the results of a pilot study including 10 patients per group. In this pilot assessment, the change in VAS score was − 3.70 ± 1.83 in patients with predominant central sensitization and − 4.85 ± 1.65 in those without predominant central sensitization. Based on these values, the between-group effect size was calculated as Cohen’s d = 0.66. Using G*Power version 3.1 software, an a priori sample size calculation was performed for an independent samples t-test with an effect size of d = 0.66, a two-sided significance level of α = 0.05, and a statistical power of 80% (1 − β = 0.80), which indicated that a minimum total sample size of 72 participants was required.

Statistical analyses were conducted using SPSS version 22 software. The normality of numerical variables was assessed through both visual methods (such as histograms and probability plots) and analytical methods (including the Kolmogorov-Smirnov and Shapiro-Wilk tests). Descriptive data were presented as the mean with standard deviation for normally distributed quantitative variables, while categorical data were summarized using frequency tables. Non-normally distributed quantitative variables were reported as medians with min-max.

For intergroup comparisons, the independent samples t-test was used for normally distributed quantitative variables, and the Mann-Whitney U test was applied for non-normally distributed quantitative variables. Categorical variables were evaluated using the chi-square test. To assess within-group differences in pre- and post-treatment outcomes, the paired samples t-test and Wilcoxon signed-rank test were used.

Time × group interaction effects on clinical outcomes were examined using linear mixed-effects models. To account for within-subject correlations due to repeated measurements, subject-specific random intercepts were included in all models. All models were adjusted for potential confounders, including age, sex, body mass index, comorbidity and main pain localization status, which were entered as fixed covariates. Time × group interaction effects were expressed as between-group differences in change over time (difference-in-differences, ΔΔ) and are reported as mean differences with 95% confidence intervals (CI) and exact p values. Effect sizes for time × group interaction effects were calculated using Cohen’s d. The significance level was set at *p* < 0.05.

## Results

**Fig. 1:**
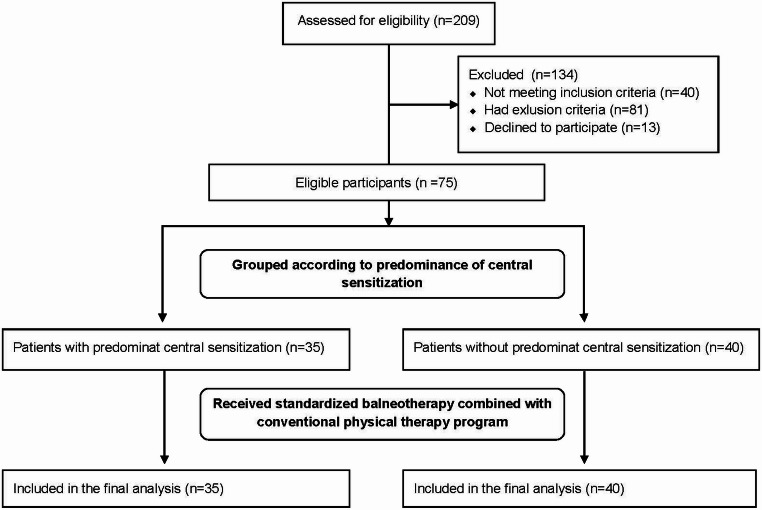
CONSORT 2010 flow diagram

A total of 209 patients were assessed for eligibility. Of these, 134 were excluded for not meeting inclusion criteria, meeting exclusion criteria, or declining participation. The remaining 75 patients (42 with chronic low back pain and 33 with chronic knee pain) were grouped according to the predominance of central sensitization and included in the final analysis (Fig. 1). Central sensitization predominance was identified in 46% (*n* = 35) of the overall sample, including 57.1% (*n* = 20) of patients with chronic low back pain and 42.9% (*n* = 15) of patients with chronic knee pain.

The mean CSI score was 57.6 ± 6.3 in participants with predominant central sensitization, whereas it was 18.1 ± 4.1 in participants without predominant central sensitization. Women were 91.4% (*n* = 32) of the predominant central sensitization group and 50% (*n* = 20) of the non-predominant central sensitization group, with a significant between-group difference (*p* < 0.001). Age, BMI, education levels, and accompanying comorbidities and diagnosis were similar between the two groups (Table 1).

**Table 1:** Sociodemographic and clinical characteristics of the participants

	Patients with predominant central sensitization *n* = 35	Patients without predominant central sensitization *n* = 40	*p* value
Age, years (median (min–max))	59 (18–76)	62 (42–78)	0.27
Gender, female, n (%)	32(91.4)	20 (50)	**< 0.001**
BMI, kg/m^2^, median (min-max)	32.5 (19.7–45.8)	28.8 (19.3–44.9)	0.20
Education level, n (%)			0.56
Illiterate	12 (34.3)	5 (12.5)	
Elementary school	13 (37.1)	13 (32.5)	
Middle school	3 (8.6)	7 (17.5)	
High school	5 (14.3)	6 (15)	
University	2 (5.7)	9 (22.5)	
Comorbidities present n (%)			0.81
Hypertension	18 (51.4)	18(45)	
Diabetes mellitus	11 (31.4)	9 (22.5)	
Hypothyroidism	4 (11.4)	4 (10)	
Coronary artery disease	3 (8.6)	4 (10)	
Main pain location, n (%)			0.85
Low back	20 (57.1)	22 (55)	
Knee	15 (42.9)	18 (45)	
Diagnoses, n (%)			0.84
Lumbar spondylosis	14 (40.0)	16 (40.0)	
Non-specific low back pain	6 (17.1)	6 (15.0)	
Knee osteoarthritis	14 (40.0)	15 (37.5)	
Meniscal pathology	1 (2.9)	3 (7.5)	

**Fig. 2:**
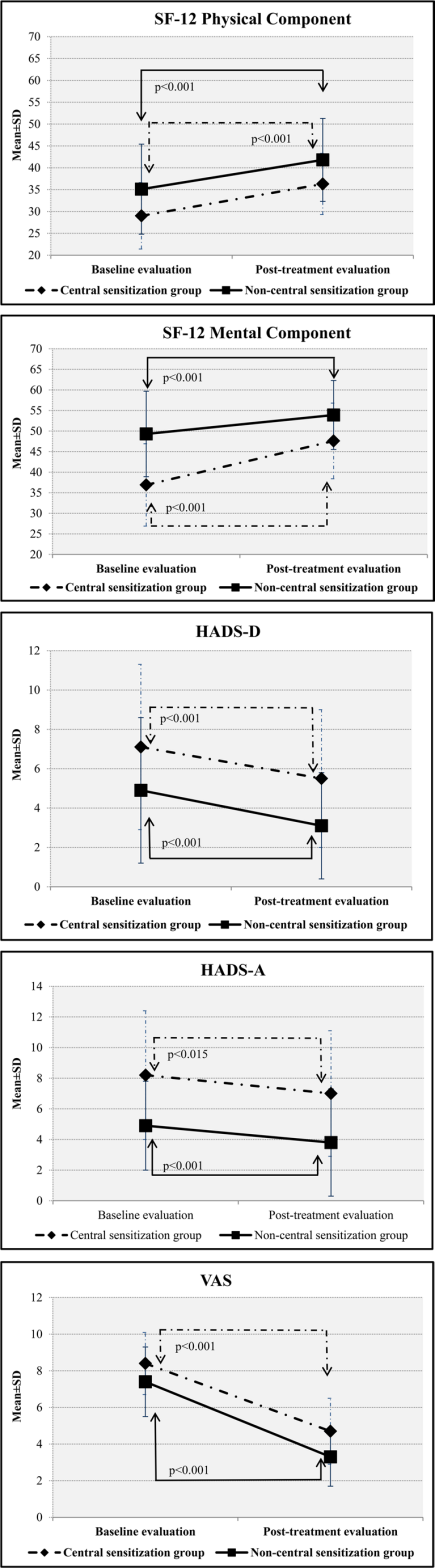
Changes in SF-12 Physical Component, SF-12 Mental Component, HADS-D, HADS-A, and VAS scores across groups

**Fig. 3:**
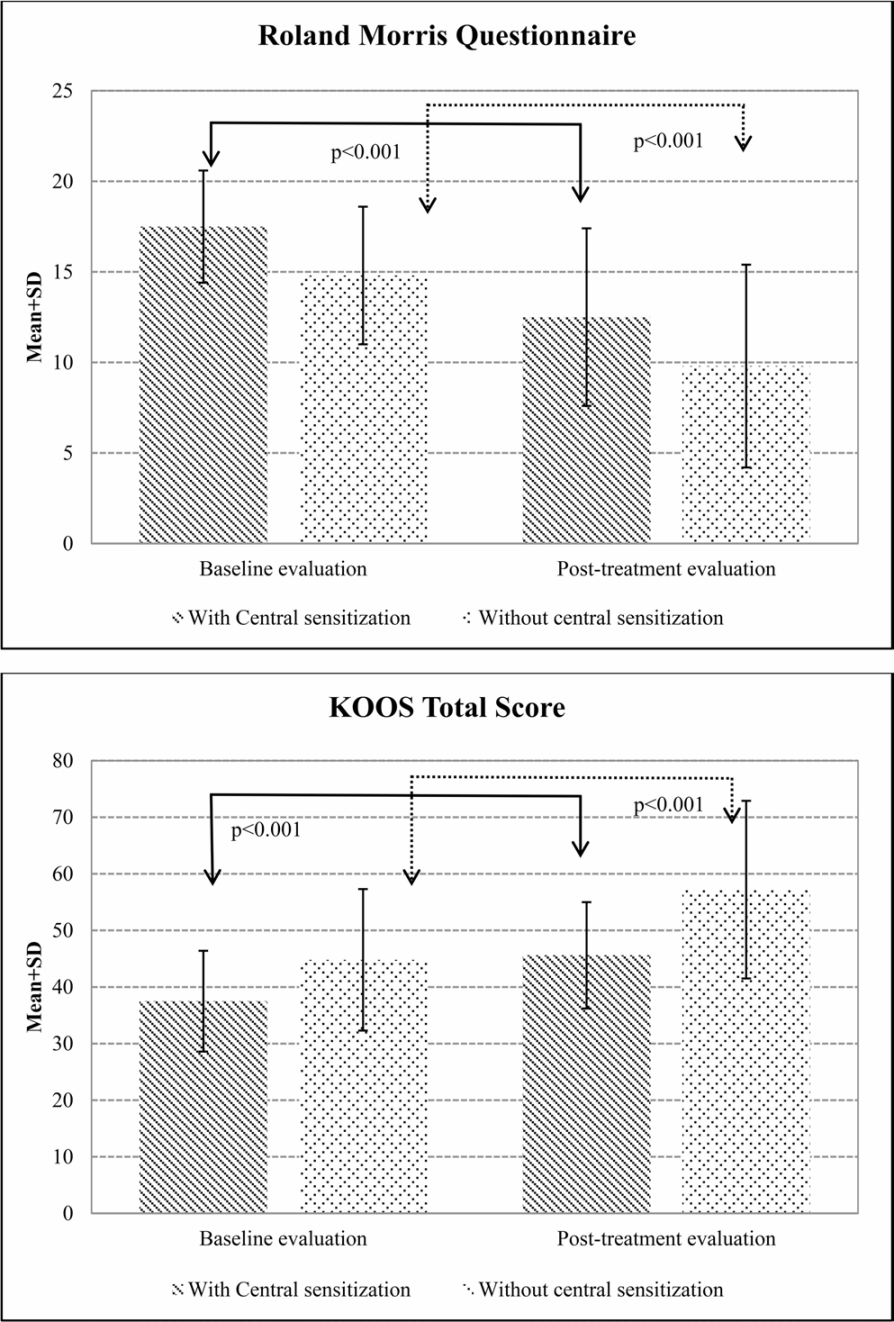
Changes in Roland-Morris Questionnaire and KOOS total scores across groups

Table 2 shows the baseline evaluations. VAS, as the primary outcome, was significantly higher in patients with predominant central sensitization (*p* = 0.02). In addition, these patients demonstrated lower SF-12 mental component scores (*p* < 0.001) and higher HADS depression and anxiety scores (*p* = 0.01 and *p* < 0.001, respectively) (Table 2). Low back pain patients with predominant central sensitization exhibited higher Roland–Morris Disability Questionnaire scores (*p* < 0.001), while knee pain patients with predominant central sensitization had lower KOOS symptoms/stiffness and quality-of-life subscale scores (*p* = 0.03 and *p* = 0.02, respectively) (Table 2) (Figs. 2 and 3).

**Table 2:** Baseline clinical outcome measures of the study groups

	Patients with predominant central sensitization	Patients without predominant central sensitization	*p* value
*All patients (n = 75)*			
VAS	8 (5–10)	7 (5–10)	0.02*
SF-12 Physical Component	27.97 (13.15–45.05)	34.7 (15.1–55.7)	0.05
SF-12 Mental Component	36.4 (18.7–65.3)	49.8 (28.6–66.7)	< 0.001*
HADS–Depression	7 (0–16)	4 (0–15)	0.01*
HADS–Anxiety	8 (1–21)	5.5 (0–11)	< 0.001*
*Patients with low back pain (n = 42)*			
Roland–Morris Disability Questionnaire	17.5 (3.1)	14.8 (3.8)	< 0.001*
*Patients with knee pain (n = 33)*			
KOOS Total Score	36 (23–54)	42 (29–74)	0.08
KOOS Symptoms and Stiffness	60.7 (15.6)	71.8 (13.5)	0.03*
KOOS Pain	44 (22–75)	50 (25–97)	0.21
KOOS Function in Daily Living	53.9 (10.8)	57.1 (13.8)	0.71
KOOS Sport and Recreation	11 (0–30)	10 (0–60)	0.59
KOOS Quality of Life	19 (0–25)	25 (0–50)	0.02*

Data are presented as median (min–max) or mean (SD), as appropriate

Baseline comparisons between groups were performed using the Mann–Whitney U test or independent samples t-test, depending on data distribution

HADS: Hospital Anxiety and Depression Scale; VAS: Visual Analogue Scale; KOOS: Knee Injury and Osteoarthritis Outcome Score.

After treatment, reductions in VAS scores were observed in both groups, along with increases in SF-12 physical and mental component scores, decreases in HADS depression and anxiety scores, and improvements in disability measures (p value between *p* < 0.001 to *p* = 0.03) (Table 3).

**Table 3:** Post-treatment outcomes results and within-group changes from baseline

	Patients with predominant central sensitization			Patients without predominant central sensitization		
	After intervention	Change from baseline	*p* value	After intervention	Change from baseline	*p* value
*All patients (n = 75)*						
VAS	5 (2–9)	−4 (− 8 − 0)	< 0.001*	3 (1–8)	−4 (− 8 − 0)	< 0.001*
SF-12 Physical Component	37.8 (17–49.4)	5.7 (− 6.8–24.4)	< 0.001*	45.5 (18.9–56.2)	4.5 (− 5.7–27.4)	< 0.001*
SF-12 Mental Component	47.6 (28.2–65.1)	10.6 (9.9)	< 0.001*	55.6 (29–64.6)	4.7 (10.2)	0.01*
HADS–Depression	6 (0–15)	−1 (− 8 − 3)	< 0.001*	3 (0–11)	−1 (− 12 − 3)	< 0.001*
HADS–Anxiety	7 (0–17)	−1 (− 10 − 4)	0.015*	3 (0–13)	−1 (− 5 − 4)	< 0.001*
*Patients with low back pain (n = 42)*						
Roland–Morris Disability Questionnaire	12.5 (4.9)	−2.8 (2.2)	< 0.001*	9.8 (5.6)	−2.7 (2.5)	< 0.001*
*Patients with knee pain (n = 33)*						
KOOS Total Score	48 (28–57)	7 (0–23)	0.002*	56 (32–83)	10 (− 1–40)	< 0.001*
KOOS Symptoms and Stiffness	70 (15.2)	9.2 (14.9)	0.03*	80.7 (11.01)	8.9 (14.6)	0.019*
KOOS Pain	61 (25–67)	11.1 (13.9)	0.009*	73.5 (39–89)	15.7 (12.4)	0.001*
KOOS Function in Daily Living	61.8 (12.4)	7.9 (11.8)	0.021*	71.8 (16.4)	14.7 (11.2)	< 0.001*
KOOS Sport and Recreation	15 (0–35)	5 (− 5–25)	0.021*	22.5 (5–85)	5 (− 15–65)	0.004*
KOOS Quality of Life	25 (0–38)	6 (− 7–25)	0.03*	31 (0–63)	6 (− 6–38)	0.003*

Data are presented as median (min–max) or mean (SD), as appropriate

Change values represent within-group changes from baseline to post-treatment.

Within-group comparisons were performed using the Wilcoxon signed-rank test or paired-samples t-test, as appropriate.

HADS: Hospital Anxiety and Depression Scale; VAS: Visual Analogue Scale; KOOS: Knee Injury and Osteoarthritis Outcome Score.

Group × time interaction effects on clinical outcomes are presented in Table 4. A statistically significant group × time interaction was observed for the SF-12 mental component score, indicating greater improvement over time in the patient with predominant central sensitization (ΔΔ = 5.95, 95% CI 1.31 to 10.59, *p* = 0.013), with a moderate effect size (Cohen’s d = 0.59). No significant group × time interaction was observed for other outcomes (range: *p* = 0.10–0.97).

**Table 4:** Group × time interaction effects for changes in clinical outcomes

	Between-group difference in change	95% Cl	*p*	Cohen’s d
*All patients (n = 75)*				
VAS	0.38	−0.43–1.19	0.35	0.22
SF-12 Physical component	0.68	−3.03–4.39	0.72	0.08
SF-12 Mental component	5.95	1.31–10.59	0.013*	0.59
HADS-D	0.09	−1.09–1.28	0.88	0.04
HADS-A	−0.13	−1.30–1.05	0.83	0.05
*Patients with low back pain (n = 42)*				
Roland Morris Disability Questionnaire	−0.02	−1.12–1.07	0.97	0.01
*Patients with knee pain (n = 33)*				
KOOS Total Score	−4.20	−8.66–0.26	0.22	0.42
KOOS Pain	−4.66	−10.76–1.45	0.32	0.35
KOOS Function – Daily Living	−6.91	−12.39 – −1.43	0.10	0.58
KOOS Function –Sport and Recreation	−8.11	−15.19 – −1.03	0.14	0.51
KOOS Synptoms and Stiffness	0.32	−10.23–10.87	0.95	0.02
KOOS Quality of Life	−2.74	−7.40–1.91	0.44	0.27

Data are presented as between-group differences in change with 95% confidence intervals.

Interaction effects were obtained from the group × time term of the mixed-effects model. Effect sizes are expressed as Cohen’s d.

HADS: Hospital Anxiety and Depression Scale; VAS: Visual Analogue Scale; KOOS: Knee Injury and Osteoarthritis Outcome Score.

## Discussion

In this prospective research, patients with chronic knee and low back pain were grouped depending on the predominance of central sensitization. The effects of balneotherapy applied together with traditional physical therapy on pain, quality of life, mood, and disability were then examined. The results revealed decreases in pain and disability and improved quality of life and mood in both groups. In addition, the improvement in the mental component of quality of life was more marked in the chronic pain with the predominant central sensitization group. To our knowledge, this study is the first to evaluate the response to balneotherapy combinate with conventional physical therapy in patients with chronic pain according to the predominance of central sensitization.

Baseline evaluation of the participants with and without predominant central sensitization revealed various striking differences. Female gender predominated in the group with predominant central sensitization. Sensitization at both the spinal and supraspinal levels has been shown to occur more easily and markedly in women (Guekos et al. 2024), and they have been reported to exhibit, under the effect of sex hormones, a greater tendency to pain catastrophization and weaker pain coping strategies (Fillingim et al. 2009). Studies have therefore shown that the prevalence of central sensitization is approximately 1.5 times higher than in men (Haruyama et al. 2021), that the central sensitization is greater among women with chronic musculoskeletal pain, and that female gender may even constitute an independent risk factor for central sensitization (Roldán-Jiménez et al. 2020; Yücel and Sanal-Toprak 2024).

Pain levels, anxiety and depression, and disabilities were all higher in the chronic pain patients with predominant central sensitization in this research. Studies have reported that even subthreshold stimuli activate the nociceptive pain pathway in the presence of central sensitization, thus increasing the level of pain experienced together with hyperalgesia and allodynia (Latremoliere and Woolf 2009), and a correlation has been determined between CSI scores and pain severity (Dahmani et al. 2023; Neogi et al. 2015). Research has reported that plasticity occurring in the prosencephalon and amygdala in response to painful stimulation during central sensitization plays a critically important role in the development of cognitive and emotional disorders including anxiety and depression, and that an association exists between them (Dahmani et al. 2023; Neugebauer et al. 2009). In addition, negative cognitive phenomena such as disease belief, fear of pain, and avoidance lead to disability by adversely affecting patients’ functional perceptions and capacities (Dahmani et al. 2023; Feuerstein and Beattie 1995).

Balneotherapy has been shown to exhibit analgesic, muscle-relaxant, and functionality-enhancing effects in patients with chronic pain (Terzic Markovic et al. 2024) as well as positive impacts on mental state, sleep quality, stress, and anxiety (Antonelli et al. 2018; Clark-Kennedy et al. 2021; Forestier et al. 2016; Nasermoaddeli and Kagamimori 2005; Tefner et al. 2012). In addition, balneotherapy in combination with traditional physical therapy is reported to yield more positive results in terms of pain severity, quality of life, and disability levels than traditional physical therapy alone (Dilekçi et al. 2020). Indeed, our participants’ pain severity decreased and their mood and disabilities improvedIn addition, this study provides novel insights by considering the predominance of central sensitization, a factor that has been largely overlooked in previous studies.

Pain levels decreased, quality of life increased, anxiety and depression levels diminished, and disability levels decreased as a result of balneotherapy applied in combination with traditional physical therapy in our chronic pain groups with and without predominant central sensitization. In addition, the decrease in SF-12 mental component scores was greater in the participants with predominant central sensitization. The limited number of previous studies considering pain sensitization in the management of chronic pain have reported that the presence of sensitization has an adverse effect on the non-pharmacological treatment of chronic pain (Yüzügüldü et al. 2023; O’Leary et al. 2018). O’Leary et al. described pain sensitization as an independent risk factor for the success of a physical therapy program based on strengthening, active range of motion, flexibility, and aerobic exercise applied to participants with knee pain. Yüzügüldü et al. applied heat, TENS, ultrasound, joint range of motion, and strengthening exercises to individuals with knee pain and showed that the presence of central sensitization was a risk factor adversely affecting pain and disability scores. Our findings are inconsistent with these results, although there are significant methodological differences between the studies, such as our application of balneotherapy and the inclusion of patients with chronic low back pain in the current research.

The effect of balneotherapy in pain management is not restricted to local mechanical effects but also involves a holistic and multi-dimensional approach with thermal, chemical, and neurophysiological components (Fioravanti et al. 2011). Mechanisms such as increased peripheral circulation, muscle relaxation, and endorphin release contribute to the amelioration of pain at the peripheral level, while hydrostatic pressure and the buoyancy characteristic of water can facilitate movement by reducing the load placed on joint and muscle groups (Gutenbrunner et al. 2010; Nasermoaddeli and Kagamimori 2005). These physical effects can lead to an increase in the patient’s functional capacity and thus to an improved quality of life. Additionally, the effects of balneotherapy on central sensitization are more complex and are to a large extent dependent on neuroimmune and neuroendocrine regulation. In particular, the application of hot water has been shown to exert a regulatory effect on autonomous nervous system activity and the hypothalamic-pituitary-adrenal axis, thus balancing cortisol levels and reducing proinflammatory cytokines (Altan et al. 2004; Bender et al. 2005). These mechanisms can contribute to reducing central nervous system oversensitivity and re-regulation of the pain threshold. Moreover, the feeling of relaxation that develops during balneotherapy, and the opportunity for patients to socialize in thermal institutions, ameliorates psychological factors linked to central sensitization, such as anxiety and depression, and this in turn mediates an improvement in both pain and quality of life (Antonelli and Donelli 2018; Tomas-Carus et al. 2008). Consistent with this, the decrease in quality of life mental scores was more marked in the central sensitization group. It may be speculated that these clinical improvements observed in this study suggest that combination balneotherapy with conventional physical therapy not only provides symptomatic relief but may at the same time produce modulatory effects on some pathophysiological processes underlying central sensitization. However, given the observational design of the study, causal inferences cannot be drawn, and further controlled and mechanistic studies with longitudinal follow-up are needed to confirm these potential effects. Also, no definitive conclusions can be drawn regarding the independent effects of balneotherapy, and these findings should be interpreted as reflecting the effects of a combined treatment approach.

There are several limitations to this study. Most notably, the lack of a control group receiving conventional physical therapy alone limits the ability to distinguish the specific effects attributable to balneotherapy. In addition, outcomes were assessed only at baseline and immediately after the intervention, precluding any conclusions regarding the durability of treatment effects over time. Another limatiton is that the study population was restricted to patients with chronic low back pain and knee pain, which limits the generalizability of the findings to other chronic pain conditions. Also, the relatively low number of male participants in the central sensitization predominant group may have limited the generalizability of the findings. Another limitation concerns the subjective nature of the questionnaire-based scales employed in the evaluation of central sensitization. Finally, because central sensitization was not assessed longitudinally, no firm causal or mechanistic conclusions can be drawn regarding the role of central sensitization in mediating pain reduction. Future research would benefit from randomized controlled designs incorporating a physical therapy–only comparator group to better delineate the independent contribution of balneotherapy. Longer follow-up periods are likewise warranted to assess the persistence of treatment effects. Studies including more diagnostically homogeneous cohorts, a more balanced sex distribution, and objective assessments such as quantitative sensory testing or neurophysiological measures may further enhance understanding of the mechanisms underlying treatment response.

## Conclusion

Balneotherapy combined with conventional physical therapy was associated with significant short-term improvements in pain intensity, physical and mental health–related quality of life, emotional status, and disability in patients with chronic low back and knee pain, irrespective of the presence of central sensitization. The magnitude of improvement was largely comparable between patients with and without predominant central sensitization, with even greater improvement observed in mental health–related quality of life among patients with predominant central sensitization. These findings suggest that the predominance of central sensitization may not substantially attenuate short-term responses to this multimodal treatment approach and may be associated with additional benefits in mental health–related outcomes. Given the observational design of the study, causal inferences cannot be drawn; therefore, further controlled studies with longer follow-up periods and mechanistic evaluations are warranted to clarify the durability of these effects and the interaction between central sensitization and treatment-related psychological and functional outcomes.

## Data Availability

The data supporting the findings of this study are available from the corresponding author upon reasonable request.
